# Codon optimisation improves the expression of *Trichoderma viride* sp. endochitinase in *Pichia pastoris*

**DOI:** 10.1038/srep03043

**Published:** 2013-10-24

**Authors:** Ping Yu, Yuan Yan, Qing Gu, Xiangyang Wang

**Affiliations:** 1 College of Food Science and Biotechnology, Zhejiang Gongshang University, 149 Jiaogong Road, Hangzhou 310035, Zhejiang Province, People's Republic of China

## Abstract

The mature cDNA of endochitinase from *Trichoderma viride* sp. was optimised based on the codon bias of *Pichia pastoris* GS115 and synthesised by successive PCR; the sequence was then transformed into *P. pastoris* GS115 via electroporation. The transformant with the fastest growth rate on YPD plates containing 4 mg/mL G418 was screened and identified. This transformant produced 23.09 U/mL of the recombinant endochitinase, a 35% increase compared to the original strain bearing the wild-type endochitinase cDNA. The recombinant endochitinase was sequentially purified by ammonia sulphate precipitation, DE-52 anion-exchange chromatography and Sephadex G-100 size-exclusion chromatography. Thin-layer chromatography indicated that the purified endochitinase could hydrolyse chito-oligomers or colloidal chitin to generate diacetyl-chitobiose (GlcNAc)_2_ as the main product. This study demonstrates (1) a means for high expression of *Trichoderma viride* sp. endochitinase in *P. pastoris* using codon optimisation and (2) the preparation of chito-oligomers using endochitinase.

Chitin comprises a large family of glycans that are composed of unbranched homopolymers of β-1,4-linked N-acetyl-glucosamine (GlcNAc), constituting a constantly recycled mass of 10^11^ tons in the biosphere per year^1^,^2^,^3^,^4^. Chitin is a major component of the cell walls of some microorganisms, including fungi, and invertebrate exoskeletons, including those of insects and crustaceans^5^,^6^,^7^,^8^. Chitin can also be degraded to functional chito-oligomers that play a role in enhancing immunity, promoting intestinal health, eliminating toxins from the body and inhibiting the growth of tumour cells^2^,^9^,^10^. The degradation of chitin currently involves acidic hydrolysis; however, some of the disadvantages, including the production of acid wastes due to the use of highly concentrated hydrochloric acid, high production costs and serious environmental pollution, cannot be easily overcome^2^. Therefore, the enzymatic degradation of chitin is gradually becoming the preferred method.

Chitin can be degraded by chitinases, which are enzymes that are generally divided into two categories: endochitinases and exochitinases^2^,^11^. Endochitinases cleave chitin polymers at random internal sites, whereas exochitinases progressively cleave chitin beginning at the non-reducing end of the chitin chain, releasing N-acetyl-D-glucosamine monomers and diacetyl-chitobiose in the process^12^. Therefore, chito-oligomers can be prepared via chitin degradation with endochitinase; however, the activity of native endochitinase from microorganisms is often low^13^,^14^. Accordingly, improving the activity of endochitinase has become a critical issue for the preparation of chito-oligomers via the enzymatic degradation of chitin.

*Pichia pastoris* is a methylotrophic microorganism in which the expression of heterologous proteins can be either constitutive or inducible. Through the use of an expression plasmid that contains an α-factor secretory signal sequence, the heterologous proteins can be secreted into the medium. To date, a large number of recombinant genes have been successfully expressed in *P. pastoris*^15^,^16^. Furthermore, several successful strategies have also been implemented to improve the expression of heterologous genes, such as increasing the copy number^17^, introducing effective transcriptional promoters^18^,^19^, optimising the culture conditions^20^,^21^ and replacing the secretory signal sequence in the expression plasmid^17^,^22^,^23^. Unfortunately, these strategies do not always result in the expected high level of recombinant protein expression^24^.

Codon optimisation is a useful technology for improving the expression of heterologous proteins. Indeed, there is a large difference between the codon usage of the host cell genome sequence and the native heterologous protein-encoding sequence, which will obviously affect the expression of recombinant proteins. Some reports have shown that the production of the target proteins was often increased an average of 1- to 5-fold by optimising the heterologous protein-encoding sequence based on the codon bias of the host cell^25^,^26^,^27^. As *P. pastoris* is not a chitinase-producing microorganism, an investigation of whether the expression of endochitinase in *P. pastoris* can be improved by codon optimisation will be of interest.

In this study, the endochitinase cDNA from *Trichoderma viride* sp. was optimised based on the codon bias of *P. pastoris* GS115, synthesised by successive PCR and successfully expressed in *P. pastoris* GS115. The expressed endochitinase hydrolysed chito-oligomers and colloidal chitin to produce diacetyl-chitobiose (GlcNAc)_2_.

## Results

### Codon optimisation of the endochitinase cDNA and its synthesis

**Figure 1:**
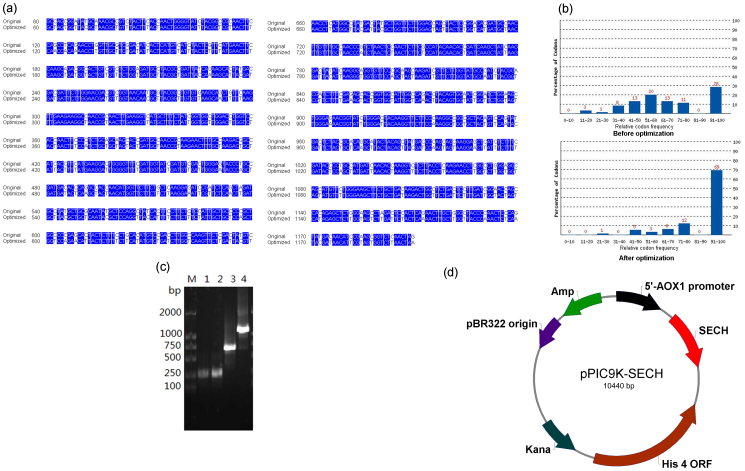
(a) Alignment of codon-optimised and wild-type endochitinase cDNAs. Bases that are the same between codon-optimised and wild-type cDNAs are marked in blue. (b) Enhanced codon usage of endochitinase for expression in *Pichia*. (c) Successive PCR of the codon-optimised endochitinase cDNA. Lane M: DL2000 marker. Lanes 1–4: Assembled PCR products of the first group of primers (F1, R1, F2, R2, F3 and R3), the second group of primers (F12, R12, F13, R13, F14 and R14), the third group of primers (F4–F11 and R4–R11) and the above three DNA fragments amplified by primer set F1 + R1, respectively. (d) Schematic map of the constructed expression vector pPIC9K-SECH.

The sequences of the wild-type and codon-optimised cDNAs were aligned, as shown in Figure 1a. Codon optimisation did not alter the amino acid sequence of endochitinase because only the third base of the codon is substituted. The codon usage of the endochitinase cDNA was optimised using the most frequently occurring triplets in *P. pastoris* GS115 such that the codon usage of endochitinase resembled that of the host strain (Fig. 1b). The synthesis of the endochitinase cDNA by successive PCR is shown in Figure 1c, with a band of the expected size (approximately 1200 bp) appearing on a 1% agarose gel after two rounds of PCR.

### Identification of the recombinant *P. pastoris* strain

**Figure 2:**
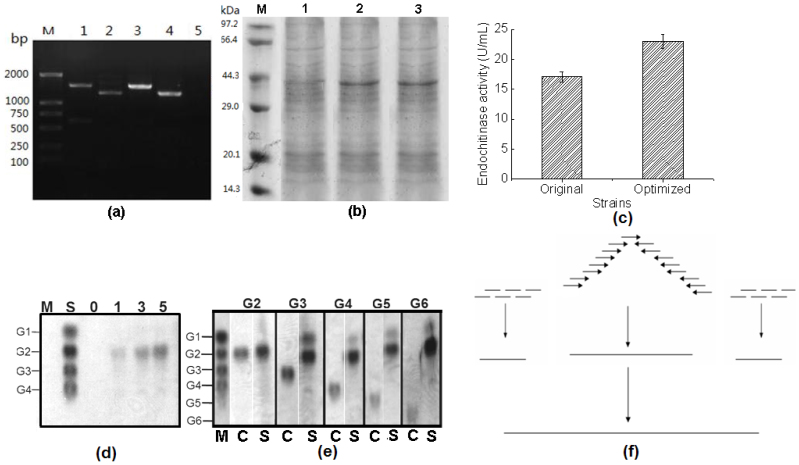
(a) Identification of the recombinant strain by PCR. Lane M: DNA marker DL2000. Lanes 1 and 3: PCR products obtained using primers 5′AOX1 and R1. Lanes 2 and 4: PCR products obtained using primers F1 and 3′AOX1. Lane 5: PCR result obtained with primers F1 and R1 using genomic DNA from the control strain as a template. (b) SDS-PAGE analysis of endochitinase expression. Lane M: Protein molecular weight standard. Lane 1: supernatant from the control strain. Lane 2: supernatant from the recombinant strain GS115 transformed with pPIC9K-ECH. Lane 3: supernatant from the recombinant strain GS115 transformed with pPIC9K-SECH. Plasmids pPIC9K-ECH and pPIC9K-SECH harbour the wild-type and codon-optimised cDNAs of endochitinase, respectively. (c) Endochitinase activities of the original and codon-optimised strains. (d) Degradable products of colloidal chitin. Lane M, the control sample without enzyme for 5 h; Lane S, standard samples (G1:G2:G3:G4 = 4:2:2:2 mM); 0, 1, 3 and 5 represent reaction times. (e) Degradable products of chito-oligomers. Lane M, standard samples (G1:G2:G3:G4:G5:G6 = 4:2:2:2:1:1 mM); Lane C, control reactions without enzyme; Lane S, endochitinase-degradable products of each chito-oligomer; G1–G6 represent (GlcNAc)_1–6_, respectively. (f) Schematic map of the assembly process of the codon-optimised endochitinase cDNAs by successive PCR.

A schematic map of the constructed plasmid, pPIC9K-SECH, is presented in Figure 1d. The codon-optimised cDNA encoding endochitinase was cloned downstream of the α-factor secretory signal sequence, which ensured the secretion of the expressed endochitinase into the medium. The results of PCR and 1% (w/v) agarose gel electrophoresis are shown in Figure 2a. The screened transformant produced bands of the expected sizes: 1293 bp (endochitinase cDNA, 1193 bp plus the 100-bp terminal sequence of 3′AOX1) or 1573 bp (endochitinase cDNA, 1193 bp plus the 380-bp terminal sequence of 5′AOX1). No bands were amplified using genomic DNA from the control strain transformed with the pPIC9K plasmid. These results indicate that linearised pPIC9K-SECH was integrated into the *P. pastoris* GS115 genome. As shown in Figure 2b, a protein band with an expected molecular weight of approximately 43 kDa was clearly apparent on the SDS-PAGE gel, indicating that the codon-optimised endochitinase cDNA was successfully expressed in the screened transformant.

### Comparison of endochitinase activity

The comparison of the endochitinase activities of the original and codon-optimised transformants (Fig. 2c) showed that the codon-optimised transformant produced 23.09 U/mL of the recombinant endochitinase, a 35% increase in comparison to the original one (17.11 U/mL). This result indicates that codon optimisation improves the activity of endochitinase.

### Purification of recombinant endochitinase

Endochitinase purification was performed sequentially using ammonium sulphate precipitation, DE-52 anion-exchange chromatography and Sephadex G-100 size-exclusion chromatography. The results of the recombinant endochitinase purification are presented in Table 1. After (NH_4_)_2_SO_4_ precipitation, 40% of the total protein was removed; the remaining proteins were subjected to DE-52 anion-exchange chromatography, resulting in a 6% yield. The protein sample was then subjected to further purification using Sephadex G-100 size-exclusion chromatography, resulting in a 2% yield. The fold purification values from the above three steps were 1.4, 5.9 and 7.9, respectively, and the recovery rates of total endochitinase activity from the above three steps were 86%, 35% and 16%, respectively.

### TLC analysis

The results for the hydrolysis of colloid chitin are presented in Figure 2d. (GlcNAc)_2_ was produced in significant amounts after hydrolysis of colloidal chitin for 1 h, and the yield of (GlcNAc)_2_ increased over the subsequent 4 h. Moreover, a small amount of GlcNAc was present after 3 h and was notable at 5 h. Chito-oligomers were also hydrolysed by the purified endochitinase, and the result is presented in Figure 2e. The recombinant endochitinase clearly did not hydrolyse (GlcNAc)_2_; however, GlcNAc was produced from (GlcNAc)_3_ after 6 h of hydrolysis. Additionally, the main hydrolytic product of the chito-oligomers was (GlcNAc)_2_ with a small amount of GlcNAc. Taken together, these results indicate that (GlcNAc)_2_ is the main degradation product of recombinant endochitinase when using chito-oligomers and colloidal chitin as the substrates.

## Discussion

Endochitinase is one of several chitinases produced by *T. viride* sp. and comprises 2–8% of its secreted proteins^28^. Glycosylated endochitinase can be produced by heterologous expression of its cDNA in the eukaryotic *P. pastoris* GS115. Although the glycosylation of endochitinase from *T. viride* sp. remains a complex issue in some microorganisms, the endochitinase produced in this study displays a good hydrolytic activity toward substrates chito-oligomers and colloidal chitin.

To improve the expression of endochitinase, differences in the relative codon frequency between *T. viride* sp. and *P. pastoris* were considered in detail. Some studies have shown that increasing the GC content of a particular gene can extend the half-life of its mRNA in *P. pastoris*^29^,^30^. However, it is also well known that a good balance of the codon frequency and GC content should be considered due to the codon bias. Based on these principles, the endochitinase cDNA from *T. viride* sp. was modified and synthesised to match the codon bias of *P. pastoris*, with a GC content similar to that in the *P. pastoris* genome. As a result, the endochitinase activity in *P. pastoris* GS115 was improved by 1.35-fold compared with the original strain. Similar results indicated that the expression of several heterologous proteins in *P. pastoris* or *E. coli* was improved by codon optimisation were reported by other researchers. Chang *et al*.^29^ reported that the expression of *Candida rugosa* lipase in *P. pastoris* was improved by 4.6-fold overall by optimising its gene sequence to match the preferred codon usage of *P. pastoris*. The gene *xynB*, encoding endo-β-1,4-xylanase from *Aspergillus sulphureus*, was synthesised by overlap extension PCR according to the codon bias of *P. pastoris*. The synthetic DNA and wild-type DNA were placed under the control of a glyceraldehyde-3-phosphate dehydrogenase (GAP) gene promoter in the constitutive expression plasmid pGAPzαA and separately electrotransformed into the *P. pastoris* X-33 strain. The maximal yield of the recombinant xylanase encoded by the synthetic DNA was 105 U/mL, which was approximately 5-fold higher than that of the xylanase encoded by the wild-type DNA under shaking-flask culture at 28°C for 3 d^31^. More interestingly, when the wild-type lipase gene (*lipJ08*) from *Candida rugosa* was expressed in *P. pastoris*, no lipase activity was detected. However, by converting 17 of the non-universal serine codons (CTG) of *lipJ08* into universal codons (TCT) by PCR-based mutagenesis, the hydrolytic activity of the recombinant LIPJ08 in *P. pastoris* was 4.7 U/mL^32^. In addition to optimising the sequence of the target gene, codon optimisation of the α-factor secretory signal sequence can improve the yield of the secreted heterologous protein. Indeed, by using the *P. pastoris*-biased secretory signal sequence (MF4I), the secreted yield of recombinant xylanase produced in shaking-flask culture was improved by 6.7-fold^33^.

The expression of chitinases from other organisms in *P. pastoris* has also been reported^34^,^35^. Endochitinase from *T. atroviride* and the class III chitinase from *Oryza sativa* can be produced at a higher yield in *P. pastoris* than in *E. coli*. Although the expression of endochitinase in *E. coli* was successfully achieved^36^,^37^, the protein expressed is often found in the form of insoluble inclusion bodies. Furthermore, attempts to restore the activity of endochitinase by refolding involve complicated and time-consuming procedures. However, the α-factor secretory signal sequence from *Saccharomyces cerevisiae* in the *P. pastoris* expression plasmid allows the expressed endochitinase to be directly secreted into the culture medium, thereby avoiding the renaturation process in addition to other benefits.

In conclusion, this study has shown that codon optimisation can improve the expression of *Trichoderma viride* sp. endochitinase in *P. pastoris*. The recombinant enzyme is produced as an active and stable biocatalyst, exhibiting good activity toward chito-oligomer and colloidal chitin substrates. These results lay a strong foundation for the industrial production of endochitinase and the preparation of chito-oligomers via chitin degradation by endochitinase.

## Methods

### Microorganisms, enzymes and chemicals

*E. coli* DH5α was purchased from Invitrogen Co. Ltd (CA, USA) and was used as the host for plasmid amplification. The Multicopy *Pichia* Expression Kit, which included *P. pastoris* GS115, pPIC9K and G418, was purchased from Invitrogen Co. Ltd (CA, USA). Pyrobest DNA polymerase, the Rapid Ligation Kit, restriction endonucleases and the DL 2000 marker were purchased from Takara Bio Inc. (Otsu, Japan). The High Pure Plasmid Isolation Kit was purchased from Roche Co. Ltd (Mannheim, Germany). DE-52, Sephadex G-100, chitin and chito-oligomers (GlcNAc)_1–6_ were purchased from Sigma-Aldrich Co. Ltd (MO, USA). All other chemicals used in the experiments were of analytical grade, and routine methods were used.

### Codon optimisation and endochitinase cDNA synthesis

To obtain mature endochitinase, 35 amino acids of the putative signal peptide and leader peptide (MLGFLGKSVALLAALQATFTSASPVTANDVSVEKR) were removed. The coding region of endochitinase was optimised based on the nuclear codon bias of *P.* *pastoris* GS115 using proprietary algorithms that substitute rare codons at the transcriptional and translational levels. The 28 primer sequences listed in Table 2 were designed to synthesise the codon-optimised endochitinase cDNA by successive PCR, as described by Xiong *et al.*^38^. An *Eco*RI restriction site was attached to the 5′ end of primer F1, and a *Not*I restriction site was added to the 3′ end of primer R1 such that the codon-optimised cDNA could be easily cloned into the *P.* *pastoris* GS115 expression plasmid pPIC9K. The primer assembly process is shown in Figure 2f. To decrease the error rate of the PCR amplification, the primers were classified into three groups, and high-fidelity Pyrobest DNA polymerase was used. During the amplification, the three groups of primer sequences were separately added to PCR reactions to produce three DNA fragments. Aliquots of the resulting DNA fragments were mixed with a primer set, F1 and R1, to amplify the full-length codon-optimised cDNA of endochitinase. The final assembled product (*sech*) was confirmed by PCR and DNA sequencing. Base mutations in *sech* were corrected by Sangon, Co. Ltd (Shanghai, China) until all the bases were well-matched with the designed sequence.

### Construction of the expression plasmid and *Pichia* transformation

The final PCR product was purified, digested with *Eco*RI and *Not*I and ligated into *Eco*RI-*Not*I-digested pPIC9K to construct the plasmid pPIC9K-SECH, and then was transformed into *E. coli* DH5α competent cells by CaCl_2_-heat shock^39^. The recombinant plasmid was isolated from the positive transformant using the High Pure Plasmid Isolation Kit. The presence and correct orientation of the insert sequence were confirmed by DNA sequencing.

pPIC9K-SECH was linearised with *Bpu*1102I for transformation into *P. pastoris* GS115. This process was used to achieve a stable integration event of one or multiple copies of the linearised plasmid at the 5′ AOX1 chromosomal locus of *P. pastoris* GS115 by homologous recombination. Competent *P. pastoris* GS115 cells were prepared by combining chemical transformation with electroporation. Approximately 1 μg of linearised plasmid was mixed with competent GS115 cells; the mixture was immediately transferred to a 0.2-cm pre-chilled electroporation cuvette and incubated on ice for 5 min. The electroporation was performed under the following conditions: charging voltage of 1.5 kV, capacitance of 25 μF and resistance of 200 ΩX. A 1-mL aliquot of 1 M ice-cold sorbitol was immediately added to the cuvette after electroporation, and the mixture was spread onto YPD plates containing different concentrations of G418 (0.5, 1.00, 2.00 or 4.00 mg/mL). The plates were incubated at 30°C until a single colony appeared. The transformant with the fastest growth rate on YPD plates containing the highest concentration of G418 was screened and identified, and its genomic DNA was isolated according to the specifications of the Multicopy *Pichia* Expression Kit. PCR amplification was performed to confirm whether the codon-optimised cDNA of endochitinase was integrated into the *P. pastoris* GS115 genome, according to the specifications of the Multicopy *Pichia* Expression Kit. The primers used, 5′ AOX1 (5′-GACTGGTTCCAATTGACAGC-3′) and 3′ AOX1 (5′-GCAAATGGCATTCTGACATCC-3′), were provided by the manufacturer (Invitrogen, USA). To compare the endochitinase activity, a transformant bearing the wide-type endochitinase cDNA of was obtained using the same procedure.

### Expression of recombinant endochitinase

The transformant was inoculated into a 250-mL baffled Erlenmeyer flask with 50 mL of buffered minimal glycerol medium (BMGY, 100 mM potassium phosphate, pH 6.0, 1.34% yeast nitrogen base (YNB), 4 × 10^−5^% biotin and 1% glycerol) and was grown at 30°C overnight with vigorous agitation at 250 rpm. When the optical density (OD_600_) reached 5.0, the cells were collected by centrifugation at 12,000 rpm for 10 min. The cell pellet was resuspended in BMMY medium (BMGY with 0.5% methanol instead of 1% glycerol) at a starting OD_600_ of 30. Methanol, as an inducer, was added to a final concentration of 0.5% every 24 h. Samples were collected for analysis after 48 h.

### Determination of the endochitinase activity and SDS-PAGE analysis

Colloidal chitin was used as the substrate for the determination of the endochitinase activity and was prepared as described by Sandhya *et al*.^40^. The reaction mixture contained 0.5 mL of enzyme, 0.5 mL of 0.5% colloidal chitin and 1.0 mL of citrate-phosphate buffer (pH 5.6). The mixture was incubated in a water bath at 40°C for 1 h; the reaction was halted by the addition of 3 mL of dinitrosalicylic acid followed by heating for 10 min. The coloured solution was then centrifuged at 12,000 rpm for 5 min, and the absorbance of the supernatant was measured at 575 nm. One unit of endochitinase activity was defined as the amount of enzyme that produces 1 μg of N-acetyl-D-glucosamine per minute at pH 5.6 and 40°C. The fermented broth of *P. pastoris* was centrifuged at 12,000 rpm for 10 min to sediment the cells, and the supernatant was subjected to 15% SDS-PAGE analysis, as described by Laemmli^41^. The resolved proteins were visualised by staining with Coomassie Brilliant Blue R250, and the total protein concentration was determined using the Bradford reagent^42^.

### Purification of the recombinant endochitinase

Unless otherwise stated, all purification steps were performed at 4°C. Cultures of *P. pastoris* bearing the codon-optimised endochitinase cDNA were centrifuged at 12,000 rpm for 20 min. Ammonium sulphate was added to the supernatant to 40% saturation, and the resulting precipitate was removed by centrifugation at 10,000 rpm for 30 min. The proteins were then fractioned by adding ammonium sulphate to different degrees of saturation (50%, 60%, 70% and 80%), and the precipitates of each were collected, combined and resuspended in 0.1 M phosphate buffer (pH 5.6). The combined precipitate was desalted by dialysis and subsequently loaded onto a DEAE-cellulose (DE-52) anion-exchange column (1.0 cm × 50 cm). The proteins were eluted at 0.8 mL/min using phosphate buffer (pH 5.8) and a linear NaCl gradient with concentrations ranging from 0 to 1 M. The fractions with endochitinase activity were collected, combined and loaded onto a Sephadex G-100 chromatography column (2.5 cm × 35 cm) pre-equilibrated with sodium phosphate buffer (pH 6.4). The proteins were eluted at 0.5 mL/min using the same buffer. The fractions with endochitinase activity were collected, combined and freeze-dried.

### Thin-layer chromatography

The purified endochitinase was used to hydrolyse the following substrates: colloidal chitin; 2 mM N-acetyl-D- glucosamine (GlcNAc); 2 mM N,N′-diacetyl-chitobiose (GlcNAc)_2_; 2 mM N,N′,N″-triacetyl-chitotriose (GlcNAc)_3_; 2 mM N,N′,N″,N′″-tetraacetyl-chitotetraose (GlcNAc)_4_; 1.5 mM penta-N-acetylchitopentaose (GlcNAc)_5_; and 1.5 mM hexa-N-acetylchitohexaose (GlcNAc)_6_. Colloidal chitin (800 μg) was incubated with 30 ng of endochitinase at 30°C in a 0.2-mL mixture containing 30 mM sodium phosphate buffer (pH 5.6) for 0, 1, 3 or 5 h. Each chito-oligomer was subjected to the same treatment as colloidal chitin except that the incubation times were changed to 0, 1, 2 or 6 h. Samples without endochitinase were used as controls. The reaction was halted by adding 0.2 mL of 0.4 M sodium carbonate buffer (pH 9.6). Aliquots (5 μL) of the reaction mixtures were separated on silica gel 60 F254S plates using a solvent system of 2-propanol:water:ammonia (34:15:1). The plates were sprayed with aniline-phthalate solution (2 mM aniline and 3.3% phthalic acid in water-saturated butanol) and subsequently heated to 150°C until dry.

## Author Contributions

Professor Y.P. conceived and designed the experiments and wrote the manuscript. The experiments were performed by Y.Y., G.Q. and W.X.Y. All authors reviewed the final manuscript.

## Figures and Tables

**Table 1:** Recombinant endochitinase purification results

Purification step	Total volume (mL)	Total protein (mg)	Total activity (U)	Specific activity (U/mg)	Purification fold	Yield (%)
Culture broth	30	77	693	9.0	1.0	100
(NH_4_)_2_SO_4_ precipitation	12	46	598	13	1.4	86
DE-52 anion-exchange chromatography	2.6	4.6	244	53	5.9	35
Sephadex G-100 size-exclusion chromatography	1.0	1.6	113	71	7.9	16

**Table 2:** Primer sequences used for synthesising the codon-optimised endochitinase cDNA

Primer name	Sequence
F1	5′-GGGCCCGAATTCGCTAGTGGTTACGCTAACGCTGTTTACTTTACTAACTGGGGTATTTACGGT-3′
F2	5′-ACTGGGGTATTTACGGTCGTAACTTTCAACCACAAAACCTTGTTGCTTCTGATATTACT-3′
F3	5′-TGTTGCTTCTGATATTACTCATGTTATTTACTCTTTTATGAACTTTCAAGCTGATGGTACT-3′
F4	5′-TTCAAGCTGATGGTACTGTTGTTTCTGGTGATGCTTACGCTGATTACCAAAAGCATTAC-3′
F5	5′-ATTACCAAAAGCATTACGATGATGATTCTTGGAACGATGTTGGTAACAACGCTTACGGT-3′
F6	5′-GTAACAACGCTTACGGTTGTGTTAAGCAACTTTTTAAGTTGAAGAAGGCTAACCGTAAC-3′
F7	5′-AGAAGGCTAACCGTAACTTGAAGGTTATGCTTTCTATTGGTGGTTGGACTTGGTCTACT-3′
F8	5′-GTTGGACTTGGTCTACTAACTTTCCATCTGCTGCTAGTACTGATGCTAACCGTAAGAAC-3′
F9	5′-ATGCTAACCGTAAGAACTTTGCTAAGACTGCTATTACTTTTATGAAGGATTGGGGTTTT-3′
F10	5′-TGAAGGATTGGGGTTTTGATGGTATTGATGTTGATTGGGAATACCCAGCTGATGATACT-3′
F11	5′-ACCCAGCTGATGATACTCAAGCTACTAACATGGTTCTTCTTCTTAAGGAAATTCGTTCT-3′
F12	5′-TTAAGGAAATTCGTTCTCAACTTGATGCTTACGCTGCTCAATACGCTCCAGGTTACCAT-3′
F13	5′-ACGCTCCAGGTTACCATTTTCTTCTTTCTATTGCTGCTCCAGCTGGTCCAGAACATTAC-3′
F14	5′-CAGCTGGTCCAGAACATTACTCTTTTCTTCATATGTCTGATCTTGGTCAAGTTCTTGAT-3′
R1	5′-GGGCCCAGCGGCCGCTTAGTTAAGACCACTACGAATGTTATCGTATTGAGAGTT-3′
R2	5′-TTATCGTATTGAGAGTTTGGGTAACCAAGCAAGTTTTGAGTAGAATCAAGACTACCAAG-3′
R3	5′-GAATCAAGACTACCAAGAGCTCTATGACTAGTACCAATCAAAGAATCAGAACCAGTCTT-3′
R4	5′-GAATCAGAACCAGTCTTATCAGCAGAAGCTTCCCAAAACATACTACCACCAAGACCAAG-3′
R5	5′-CTACCACCAAGACCAAGGTTCTTAAGGTAAGAAACCTTAGTGTTAATCATAGCTGGAGT-3′
R6	5′-TTAATCATAGCTGGAGTATCAAAAGAAATAAGTTCCTTACTACTTGGATCGTAACTGTA-3′
R7	5′-CTTGGATCGTAACTGTAGTAAGCTTGAGCAGTAGAATCGTATTGAACAGTAGCACCAGC-3′
R8	5′-TGAACAGTAGCACCAGCCTTTGGAAGAACCTTGTAATCCCAAATACCGTTTTCCCAACT-3′
R9	5′-ATACCGTTTTCCCAACTACCAGAACCAATACCACTGTAAGTTTGACCAATACCACCAGT-3′
R10	5′-TGACCAATACCACCAGTACTTTCAAAAGAACGACCGTAAATTGGCATACCAAGAACAAT-3′
R11	5′-GGCATACCAAGAACAATCTTACTAGCTGGAACACCACCCTTAATGTAATCCTTAATAGC-3′
R12	5′-ATGTAATCCTTAATAGCTTGATCAGTGTTGTATGGAGAAGAGTTAGAGTTAGATGGGTT-3′
R13	5′-TTAGAGTTAGATGGGTTAGCAAACAAGTTAGCATCATGACCAGAGTAACTACTCCAAGA-3′
R14	5′-GAGTAACTACTCCAAGAACCAGCGTAATCGTAAGCCATAAGGTTAACGTAATCAAGAACTTGACCAA-3′

The underlined letters in F1 and R1 are the *EcoR*I and *Not*I restriction sites, respectively.
